# Effect of Closed-Loop Vibration Stimulation on Heart Rhythm during Naps

**DOI:** 10.3390/s19194136

**Published:** 2019-09-24

**Authors:** Sang Ho Choi, Heenam Yoon, Hyung Won Jin, Hyun Bin Kwon, Seong Min Oh, Yu Jin Lee, Kwang Suk Park

**Affiliations:** 1 Interdisciplinary Program in Bioengineering, Graduate School, Seoul National University, Seoul 03080, Korea; csh412@bmsil.snu.ac.kr (S.H.C.); jinhyungwon@bmsil.snu.ac.kr (H.W.J.); chasekwon@bmsil.snu.ac.kr (H.B.K.); 2 Artificial Intelligence Laboratory, Software Center, LG Electronics, Seoul 06772, Korea; hnyoon@bmsil.snu.ac.kr; 3 Institute of Medical and Biological Engineering, Medical Research Center, Seoul National University, Seoul 03080, Korea; 4 Department of Neuropsychiatry and Center for Sleep and Chronobiology, Seoul National University Hospital, Seoul 03080, Korea; nightwing@snu.ac.kr (S.M.O.); ewpsyche@snu.ac.kr (Y.J.L.); 5 Department of Biomedical Engineering, College of Medicine, Seoul National University, Seoul 03080, Korea

**Keywords:** closed-loop system, vibration stimulation, interaction, heart rhythm, nap

## Abstract

Sleep plays a primary function for health and sustains physical and cognitive performance. Although various stimulation systems for enhancing sleep have been developed, they are difficult to use on a long-term basis. This paper proposes a novel stimulation system and confirms its feasibility for sleep. Specifically, in this study, a closed-loop vibration stimulation system that detects the heart rate (HR) and applies −n% stimulus beats per minute (BPM) computed on the basis of the previous 5 min of HR data was developed. Ten subjects participated in the evaluation experiment, in which they took a nap for approximately 90 min. The experiment comprised one baseline and three stimulation conditions. HR variability analysis showed that the normalized low frequency (LF) and LF/high frequency (HF) parameters significantly decreased compared to the baseline condition, while the normalized HF parameter significantly increased under the −3% stimulation condition. In addition, the HR density around the stimulus BPM significantly increased under the −3% stimulation condition. The results confirm that the proposed stimulation system could influence heart rhythm and stabilize the autonomic nervous system. This study thus provides a new stimulation approach to enhance the quality of sleep and has the potential for enhancing health levels through sleep manipulation.

## 1. Introduction

Humans sleep almost one-third of their lifetimes. Sleep plays an important role in our lives in terms of health and well-being. Therefore, monitoring sleep and enhancing its quality are important for leading a healthy life. Several smart technologies have been developed to monitor sleep in the typical home environment [1]. However, although such methods provide the user with sleep information, methods that extend beyond the passive monitoring of sleep are required to enhance sleep quality and promote health levels through sleep. To enhance sleep, soothing sounds or music and feet warming are commonly used among the general population [2,3]. In addition, rocking movements appear to help people relax or fall asleep. Swinging a baby in a hammock or physical rocking movements can be helpful in inducing sleep and appear to be effective for adults as well [4,5,6,7].

Several methods have been developed to increase sleep efficacy by enhancing the slow wave activity (SWA). Electroencephalogram (EEG) SWA, which represents the EEG spectral power in the 0.5–4 Hz band during non-rapid eye movement (NREM) sleep, is an important contributor to memory consolidation and brain restoration [8]. Intermittent transcranial direct-current stimulation increases slow wave sleep (SWS) and the <1 Hz slow oscillation during stimulation-free intervals [9]. Another study demonstrated that slow waves can be triggered in sleeping subjects with transcranial magnetic stimulation [10]. An auditory stimulation method has been shown to enhance slow oscillations [11]. Recently, many studies that use these methods to enhance brain oscillations and improve sleep and memory consolidation have been conducted [12,13,14,15,16]. However, although these methods affect sleep, their safety is questionable, and they are considered impractical for long-term use. Thus, other stimulation systems for enhancing sleep quality are needed.

In many natural phenomena, oscillating objects with their own rhythm interact with the environment [17]; for example, thousands of fireflies blinking on and off in unison. Fireflies interact with other insects via light pulses, and each firefly is affected by the light created by the entire population [18]. A cricket’s chirps are influenced by the chirps of its neighbors. A cricket responds to the preceding chirp and achieves synchrony by either lengthening or shortening its chirp [19]. Moreover, interactions are also present in human physiological systems; there are interactions in human internal subsystems, such as respiratory sinus arrhythmia (RSA), which refers to the periodic variation in the heart rate (HR) according to the respiratory cycle [20]. In addition, the cardiac system interacts with brain activity [21,22] and locomotor rhythms [23,24].

Furthermore, the internal physiological system is influenced by environmental conditions and change. The circadian rhythm represents the behavior of humans with a 24 h cycle of sleep and wakefulness. This cycle is entrained by the daily cycle of light and dark [25,26]. McClintock reported that social interaction influences some aspects of the menstrual cycle [27]. Van Leeuwen et al. [28] verified phase synchronization, which implies the existence of phase locking between two weakly interacting systems, such as between the fetal and the maternal HRs, even though they are part of autonomic nervous systems (ANS) with separate blood circulation. They stated that maternal–fetal heart coupling is mediated by the acoustic stimulation of maternal heartbeat and vascular pulsations, which are recognized by the fetal auditory system. These stimuli can act on external forced rhythms to synchronize the heartbeat of the fetus with that of the mother. Grimaldi et al. [29] were the first to prove that the acoustic enhancement of SWA during sleep enhances parasympathetic activity. They stated that acoustic stimulation strengthened the coupling between cortical and cardiac oscillations, which was reflected in the concomitant changes in SWA and heart rate variability (HRV). A study that assessed the interaction between an internal physiological system and external forces showed that the HR can be entrained through a weak external noninvasive force in the form of visual and auditory stimuli [30]. In our previous study [31], we found experimental evidence that couples’ cardiac rhythms influence each other during co-sleeping. This finding may be attributed to weak cardiac vibrations that are transmitted from one individual to another through a mechanical bed connection.

These studies showed that the intrinsic physiological rhythm could be entrained and interact with the periodic rhythm of other systems in at least one neural, mechanical, or behavioral connection. Experiments were conducted during naps; hence, the effects of other external factors on the physiological system were minimized when the subjects were sleeping relative to when they were awake. In a normal human sleep pattern [32], NREM and REM sleep alternate through the night in approximately 90-min cycles. HR decreases progressively from the sleep onset period to the deeper NREM sleep and increases during REM sleep. Furthermore, in general, the human internal physiology systems have been well researched during sleep. Therefore, we anticipated that the effects of stimulation could be examined more accurately during sleep.

In this study, we developed a novel closed-loop vibration stimulation system and investigated the effect of stimulation on heart rhythm. We hypothesized that an external weak vibration stimulus could influence heart rhythm and stabilize the ANS during sleep. Furthermore, if detuning, which represents the frequency difference between an oscillator and an external force, is small, even a very small force can entrain the oscillator [17]. Thus, we hypothesized that a smaller amount of detuning is appropriate for modulating heart rhythm.

## 2. Materials and Methods

### 2.1. Closed-Loop Vibration System

**Figure 1:**
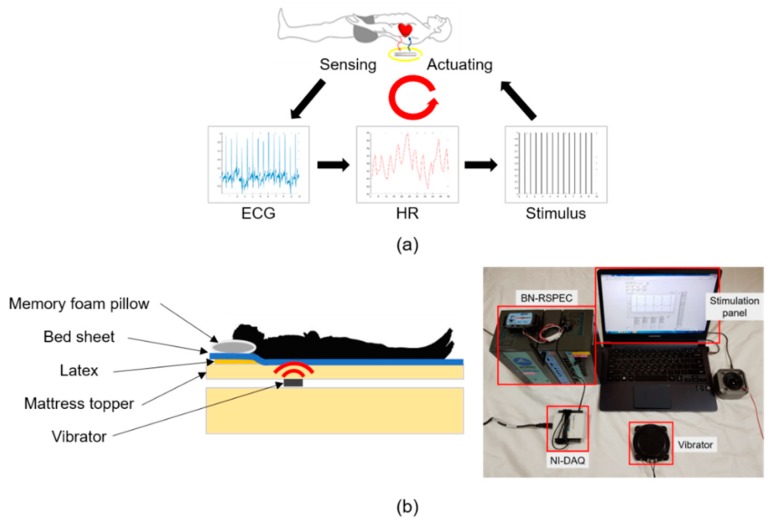
(**a**) Developed system consisting of ECG recording, HR detection, and stimulus actuation. These processes are repeated in a closed-loop manner. (**b**) Closed-loop vibration system diagram and devices used in the experiment. ECG—electrocardiogram; HR—heart rate.

**Figure 2:**
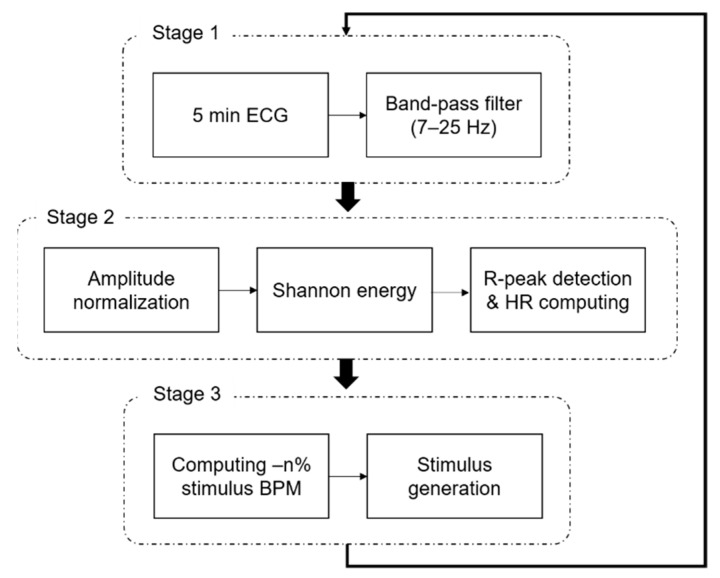
Block diagram of the closed-loop processes. BPM—beats per minute.

Figure 1 and Figure 2 show our experimental system and a block diagram of the closed-loop three-stage processes, respectively. Stage 1 includes the measurement of the electrocardiogram (ECG) signal and a band-pass digital filter. The ECG signal was recorded using a wireless device (BN-RSPEC; Biopac Systems, Inc., Goleta, CA, USA) at the lead 2 position, and the sampling rate was set to 500 Hz. Then, the ECG signal was filtered between 7 and 25 Hz. In stage 2, the real-time HR was computed from the ECG signal. In this study, the ECG R-peak, which represents the dominant peak of the QRS complex, detection method based on the Shannon entropy was applied [33]. The filtered ECG signal was normalized, and the Shannon entropy was computed. If the Shannon entropy of a sample was higher than the threshold value, that sample was considered as the R-peak. The HR was calculated from the difference in the R-peak indices; then, the mean HR was computed every 5 min. The mean and standard deviation of absolute errors between the HR extracted from the R-peak using the automatic algorithm [34] (and corrected manually) and the real-time HR were 0.17 and 1.61 bpm, respectively. Furthermore, the real-time R-peak detection accuracy was 99.5%, which is an acceptable level of performance for a real-time peak detection algorithm. In stage 3, the value of −n% stimulus beats per minute (BPM) was computed on the basis of the mean HR calculated over the previous 5 min, and a vibration stimulus was generated. We hypothesized that an external stimulus with a rate lower than the HR could decrease the heart rhythm rate. Subsequently, an experiment considering stimulation conditions of −3%, −5%, and −10% in frequency was conducted. A woofer was used as the vibrator and installed between the mattress and the mattress topper, as shown in Figure 1b. The vibrator was positioned such that it was near the subject’s heart when the subject was lying on the bed. ECG signals were collected in real time through an NI-DAQ device (USB-6003; National Instruments, Austin, TX, USA), and a LABVIEW program (version 15.0.1, National Instruments, Austin, TX, USA) was used to compute the HR and stimulus BPM. The aforementioned three steps were repeated in a closed-loop manner, and the stimulus BPM was updated every 5 min.

### 2.2. Experimental Design and Procedure

The study was conducted in accordance with the Declaration of Helsinki, and the Institutional Review Board of Seoul National University Hospital approved this prospective cohort study (IRB No. C-1805-165-948). We recruited participants by posting leaflets on the school bulletin board. Before proceeding with the experiment, a questionnaire was collected to ensure that each participant met the inclusion and exclusion criteria of the experiment. The inclusion criteria for this study were as follows: the participant (1) had to be 18–40 years of age and (2) must be healthy with no symptoms related to sleep. The exclusion criteria for this study were as follows: people (1) with a history of severe physical or psychological illnesses, (2) suffering from arrhythmia, (3) taking medicines that affect sleep, (4) who have consumed alcohol in the three days prior to the experiment, and (5) who suffered from irregular sleep in the three days before the experiment. Ten people (six men, four women) who satisfied the inclusion and exclusion criteria participated in the experiments. All subjects were briefed about the methods and procedure of this study and signed informed consent forms. The mean and standard deviation (SD) of the subjects’ ages were 27.1 and 3.3 years, respectively (min.–max.: 22–32 years). The mean and SD of the subjects’ body mass index (BMI) were 22.2 and 2.4 kg/m^2^, respectively (min.–max.: 17.9–26.7 kg/m^2^).

Each subject participated in one baseline condition and three stimulation conditions, for which the stimulus BPM percentage was set to −3%, −5%, and −10%. To detune the rates between the HR and the weak noninvasive forcing, the ±5% stimulus was considered appropriate in a previous study [30]. We hypothesized that a negative percentage is appropriate for decreasing the HR and stabilizing the ANS. Thus, we considered the stimulus detuning conditions of −3%, −5%, and −10% in frequency. The stimulation experiments were conducted in a random order. Each experiment was conducted in an interval of at least one week. All subjects were asked to refrain from consuming alcohol for 3 days before the experiment and from consuming caffeine on the day of the experiment. They participated in the experiments after eating lunch and took a nap that was approximately 90 min long. Before conducting stimulation experiments, the intensity of the stimulus was individually adjusted in order to prevent the vibration interfering with sleep. The subjects completed questionnaires related to the subjective sleep quality or vibration stimulus after waking up from the nap.

### 2.3. Heart Rate Variability Analysis

In this study, we used three analysis methods to evaluate the effect of the closed-loop vibration system. First, we analyzed the HRV, which is an efficient, noninvasive, and unobtrusive method used to investigate the modulation of the autonomic nerve activity [35]. Before extracting the HRV parameters, the ECG signals were filtered to remove noise and baseline drift through high-pass filtering at 3 Hz and were then sequentially low-pass filtered at 30 Hz (fifth order, infinite impulse response, Butterworth). The ECG R-peaks were detected using a self-developed automatic peak detection algorithm [34] and then manually corrected.

Four time-domain parameters — specifically, HR, percentage of successive normal-to-normal (NN) intervals differing by more than 50 ms (pNN50), standard deviation of the NN intervals (SDNN), and root mean square of successive NN-interval differences (RMSSD) — were computed. Furthermore, the HRV parameters were extracted in the frequency domain. Cubic interpolation, which is a shape-preserving method, was applied to the R-R intervals; then, the spectral power was computed using a fast Fourier transform. From the spectral power, the following parameters were computed: low-frequency (LF) band power (0.04–0.15 Hz) and high-frequency (HF) band power (0.15–0.4 Hz), which were normalized by dividing by the sum of the LF and HF. In addition, the ratio of the LF power to the HF power (LF/HF) was extracted. We computed seven HRV parameters every 5 min and analyzed the difference of these parameters under the baseline and stimulation conditions.

### 2.4. Heart Rate Density Analysis

**Figure 3:**
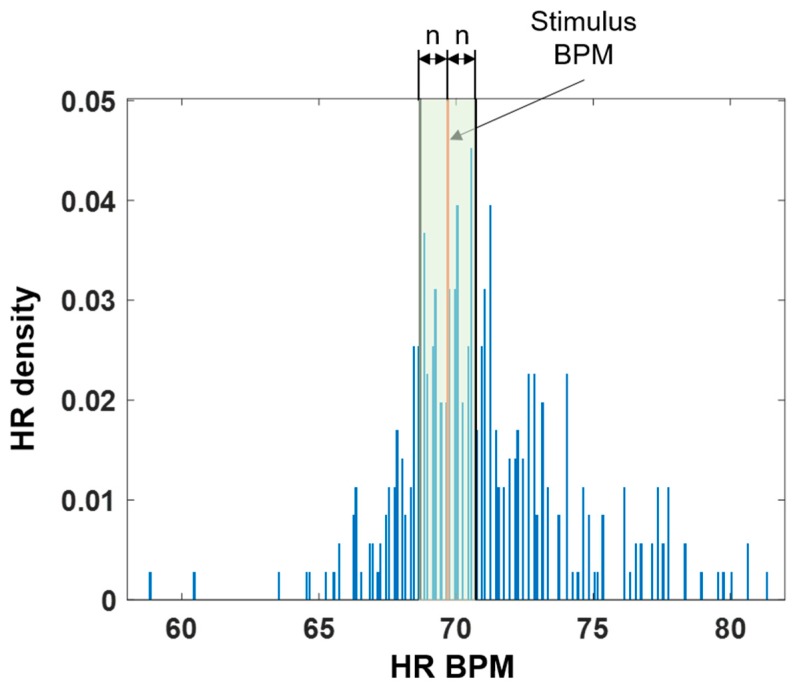
Example of a heart rate density distribution from subject 1 under the −3% stimulation condition at an n of 2.0. The stimulus BPM was computed on the basis of the mean HR calculated over the previous 5 min. Orange line: position of the stimulus BPM. Shaded green: area of the densities between stimulus BPM ± *n* BPM, *n* = 0.5, 1.0, and 2.0 BPM.

We also analyzed the HR density to check whether the HR was modulated around the stimulus BPM. A histogram was computed in 0.1 BPM intervals based on the minimum and maximum values of the recorded 5-min HR. Then, the histogram was divided by the total number of heartbeats to extract the HR density. Next, the sum of the densities within ±*n* BPM was calculated on the basis of the stimulus BPM to confirm the number of heartbeats that concentrated around it when the stimulus was applied. The green shaded area in Figure 3 shows the extracted HR density area. The values of *n* were set to 0.5, 1.0, and 2.0 BPM.

To compare the results of the baseline and stimulation tests, we required surrogate stimulus data even though there was no stimulus BPM under the baseline condition. We computed the stimulus BPM for the baseline data by using the same rule used to compute the −*n*% stimulus BPM based on the previous 5-min mean HR. Then, we compared the HR density for the surrogate and stimulus conditions.

### 2.5. Synchronization Analysis

**Figure 4:**
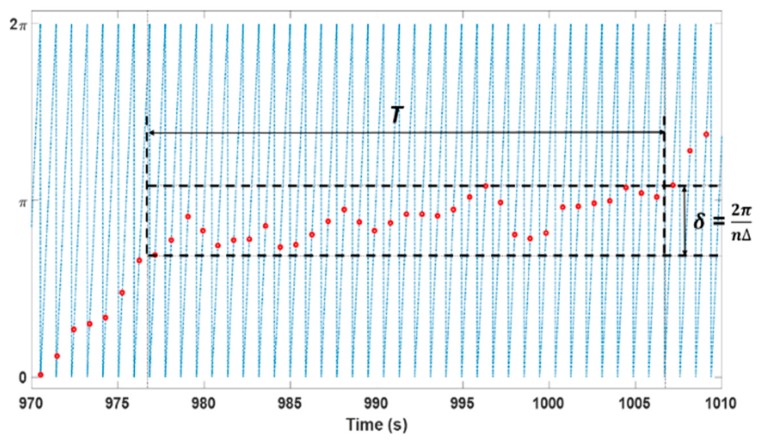
Phase synchronization and synchrogram method. Each R-peak location of the ECG (red dots) is placed at the corresponding location of the instantaneous phase on the stimulus (blue line). A synchronization epoch was determined for the segment where the variation in the points was maintained within δ = 2π/(*n*Δ) and prolonged for *T* seconds. *n*, Δ and *T* were set to 1, 5 and 30, respectively. ECG—electrocardiogram.

Finally, we analyzed the synchronization between heartbeats and stimuli. Phase synchronization analysis is a measurement of the intrinsic frequency and phase of two systems that are locked at a certain rate because of their interaction [36]. In this study, we analyzed the phase synchronization between heartbeats and stimuli by using the synchrogram method [36,37,38], which is a visualization tool used to detect the synchronization epochs between two signals. As such, phase-synchronization epochs were detected where the variation in the points was maintained within δ = 2π/(*n*Δ) and prolonged for *T* seconds, as shown in Figure 4. In our analyses, the value of Δ, which is the threshold determinant factor, was set to 5, and T, which is the standard window size for sleep analysis, was set to 30 s. We detected the synchronization epochs only under the 1:1 ratio (n = 1) condition for heartbeats: stimuli. The surrogate data were constructed from the baseline data to check the effect of vibration stimulation on synchronization. We applied the same rule by which the −*n*% stimulus BPM was calculated from the previous 5 min mean HR to obtain the stimulus signal for the baseline data. Then, the synchronization ratio was computed from the surrogate data and compared with the synchronization ratio of the stimulation data. The HRV, HR density, and synchronization were analyzed using MATLAB R2018b (MathWorks, Natick, MA, USA) software.

### 2.6. Statistical Analysis

To verify the effect of the stimulation, a Wilcoxon signed-rank sum test, a nonparametric statistical analysis, was employed because the data were not normally distributed. A p-value of less than 0.05 was considered significant. The statistical analysis was performed using the SPSS statistics program (v. 25.0, SPSS Inc., Chicago, IL, USA).

## 3. Results

### 3.1. Heart Rate Variability

**Figure 5:**
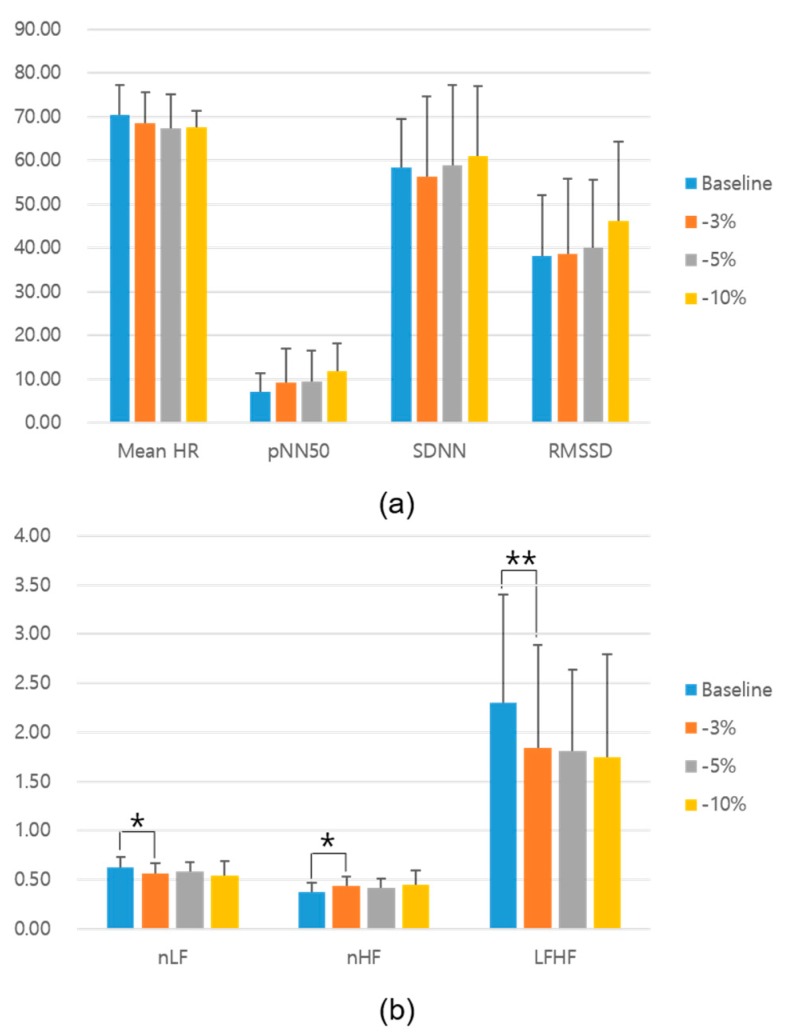
HRV parameters under each experimental condition: (**a**) time and (**b**) frequency-domain HRV results. * *p* < 0.03 and ** *p* < 0.01 between the baseline and stimulation conditions (Wilcoxon rank-sum test). HRV—heart rate variability.

Figure 5 presents the time–and frequency–domain HRV parameters according to each group. There was no significant difference in the time-domain HRV parameters for the baseline and stimulation conditions. However, in the frequency domain, the normalized LF (nLF) and LF/HF ratio parameters were significantly lower under the −3% stimulation condition than under the baseline condition (*p* < 0.03 and 0.01, Wilcoxon signed-rank sum test). In addition, the normalized HF (nHF) parameter under the −3% stimulation condition was significantly higher than that under the baseline condition (*p* < 0.03, Wilcoxon signed-rank sum test). Moreover, no significant differences were observed between the HRV parameters for the baseline and −5% or −10% conditions. Table 1 presents the average and SD of the HRV parameters according to each condition.

### 3.2. Heart Rate Density

**Figure 6:**
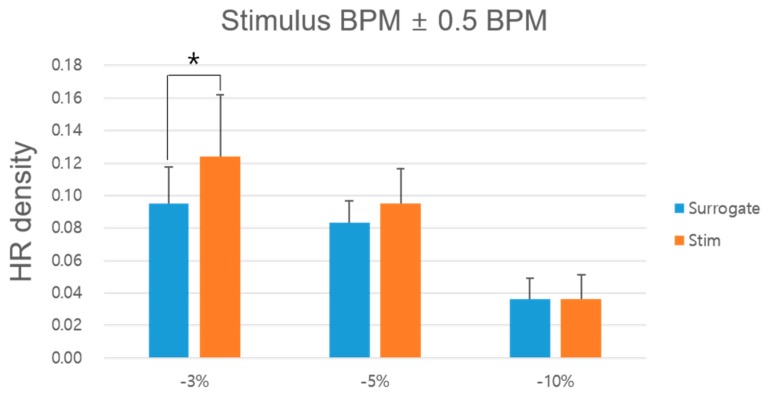
HR densities in stimulus BPM ± 0.5 BPM for each experimental condition. * *p* < 0.01 between the baseline and stimulation conditions (Wilcoxon rank-sum test). HR, heart rate.

Figure 6 shows the sum of the densities within 0.5 BPM based on the stimulus BPM. In addition, Table 2 summarizes the average of the HR densities in each interval. A significant increase was observed between the surrogate and −3% stimulus HR densities. In every interval, the HR BPM densities were significantly higher under the −3% stimulation condition than under the surrogate condition (*p* < 0.03, Wilcoxon signed-rank sum test). However, there was no significant difference between the surrogate and −5% or −10% conditions.

### 3.3. Synchronization Ratio

We computed the synchronization ratio between heartbeats and stimuli. Table 3 presents each subject’s synchronization ratio under the stimulation conditions. In general, the results exhibited an increased average of synchronization value and the number of subjects whose synchronization value was improved (7 subjects vs. 3 subjects in −3% stimulation); however, the statistical significance level was not sufficiently low to confirm the synchronization difference between the surrogate and stimulation conditions.

## 4. Discussion

**Figure 7:**
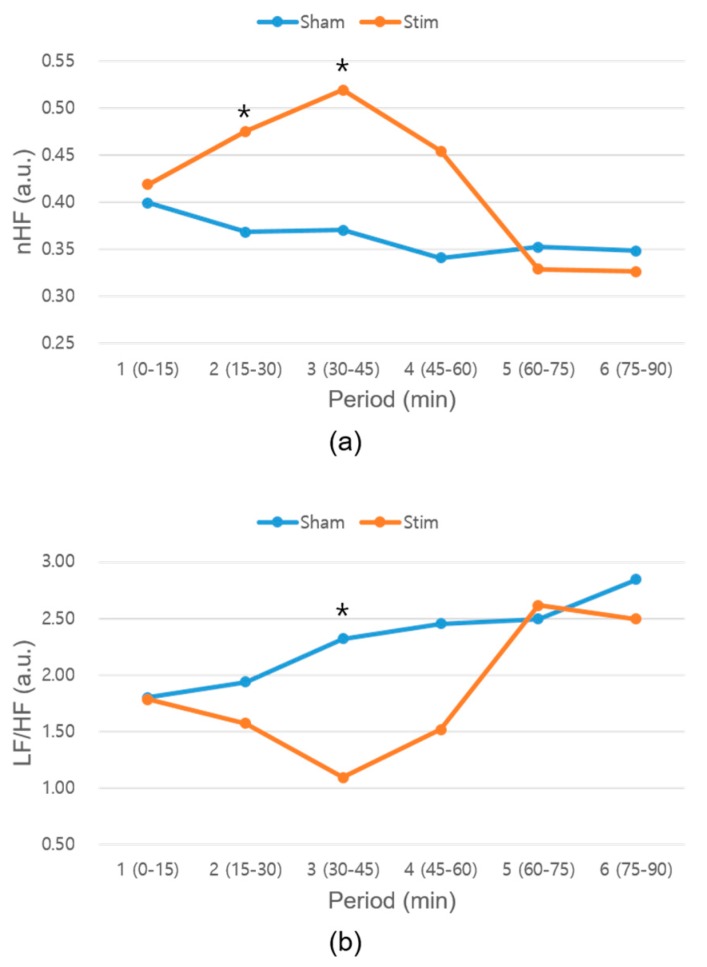
Results of the HRV extracted every 15 min under the baseline and −3% stimulation conditions: (**a**) nHF and (**b**) LF/HF parameter results. HRV—heart rate variability; nHF—normalized high-frequency band power; LF/HF—ratio of the low-frequency power to the high-frequency power.

In this study, we developed a novel closed-loop vibration stimulus system based on HR and evaluated the effect of the developed system on heart rhythm during napping. The HRV analysis confirmed a significant difference between the baseline and −3% stimulation conditions. The nHF parameter, which represents the parasympathetic activity [35], significantly increased, and the LF/HF parameter, which represents the sympathovagal balance [35], significantly decreased under the −3% stimulus condition. These results indicate that −3% stimulation makes the ANS more stable. It is possible that the effects of ANS stabilization, such as increased SWS or less sleep-stage transition, may have resulted in more stable sleep. When we analyzed the HRV every 15 min, the nHF parameter for the −3% stimulation condition significantly increased in the second and third periods (Figure 7a), and the LF/HF parameter significantly decreased in the third period (Figure 7b). The second or third period of the HRV corresponds to the SWS time, which normally occurs 20–40 min into the first cycle [32]. Thus, it is possible that the SWS was increased or that the sleep stage was stabilized under the −3% stimulation condition. Compared with the −3% stimulation condition, no significant differences were observed between the corresponding HRV parameters under the baseline and −5% or −10% conditions. Even though the mean HRs of the subjects differed under the different experimental conditions listed in Table 1, their difference was not statistically significant. Further, frequency-domain HRV parameters, one of our main results, under baseline and stimulation conditions were compared after normalization. While the HRs of the subjects were different, we detected the mean HR every 5 min and applied an n% lower BPM stimulus based on the previous 5-min mean HR. We adopted the closed loop stimulation for this study to reflect the temporal HR variation in real time and minimize the effect of daily mean HR difference. The most appropriate way to conduct stimulations was based on the previous mean HRs, as the significance of the stimulation effect did not deteriorate even if the subjects had different mean HRs on different days.

In the HR BPM density analysis, the density significantly increased in all intervals under the −3% stimulation condition compared with the surrogate data, which were extracted from the baseline data. This implies that the closed-loop vibration system affected the shifting of the heart rhythm around the external stimulus BPM. Furthermore, no significant differences were observed between the surrogate data and the −5% or −10% conditions. Therefore, the −3% stimulation condition was appropriate for modulating the heart rhythm, and it could be said that an external stimulus closer to the HR had a larger effect on the HR modulation. According to a previous study [30], the ±5% stimulation range is appropriate for detuning between the HR and weak noninvasive forcing. In the current study, we only tested the negative percentage conditions to stabilize the heart rhythm. When an external weak stimulation is applied to the heart, which is a self-sustained oscillator, a smaller phase difference is more suitable for modulation. In our experiment, a −3% stimulation was more appropriate than the other values for modulating the heart rhythm.

The synchronization analysis showed no significant differences between the surrogate and stimulation data. Although no statistical difference was observed for the subject-specific synchronization rate, we found that the synchronization ratios of seven subjects increased under the −3% stimulation condition (Table 3). However, the synchronization ratio of five and two subjects was observed to increase under the −5% and −10% simulation conditions, respectively, compared to the surrogate data. We expected that if our developed system affected heart rhythm, the HR density and synchronization would be changed. Although the HR densities were significantly increased, the synchronization ratios did not increase in a statistically significant manner. This was because the synchronization analyzes the phase-lock period, which lasted more than *T* seconds. Although increasing the HR density did not always lead to an increase in the synchronization ratio, there was a significant positive correlation between the HR density and the synchronization ratio (Pearson’s correlation coefficient = 0.762, *p* < 0.01). Because the results exhibited a tendency toward synchronization, further studies are required with a targeted experimental setup and an increased number of subjects.

In summary, the closed-loop vibration-stimulation system effected the change in the HR density and the stabilization of the ANS. Specifically, −3% stimulation was more appropriate for modulating heart rhythm than the −5% and −10% cases. Human physiological systems interact with internal subsystems or external systems. Specifically, the rhythm of the cardiac system could be entrained by external weak forcing [30]. Human heart rhythms synchronize, while co-sleeping and the heart rhythm of one co-sleeper can act as an external stimulus that affects the heart rhythm of the other co-sleeper [31]. The results of our study may be attributed to the independent and weak but continuous vibration rhythm system interacting with the cardiac system. Existing stimulation methods for sleep enhancement [9,10,11,12,13,14,15,16] could be inconvenient for long-term use, while our system has the advantage of unobtrusive stimulation. As shown in Table 4, for the questions related to the discomfort of the stimulation system, no significant differences were observed between the baseline and stimulation conditions. If we detect the HR through the ballistocardiogram (BCG) signal by using a sheet-type sensor, such as an EmFit or polyvinylidene fluoride sensor, which can be unobtrusively installed under the bed sheet, our system could reduce the hassle of attaching the sensor and can comprise a closed stimulating loop in an unobtrusive or unconstrained manner. Therefore, our system could be a new method for applying external stimulation during sleep. In addition, brain computer interfaces, initially developed to translate brain activity and communicate with the environment without limb movement, could also be applied to assess cognitive abilities [39,40]. In this manner, our system could be used in other fields. Sleep is associated with memory and good sleep quality improves memory consolidation. If a vibration stimulus enhances sleep quality and memory consolidation, our system could be applicable to improve cognitive ability. We will confirm the possibility of improving memory consolidation during sleep in a future study.

The aims of this study were to propose a new system and investigate the effect of stimulation on heart rhythm. However, there are some limitations. First, we evaluated the proposed system with 10 subjects and checked the possibility of modulating their heart rhythms during napping. More subjects are needed to evaluate the system during a whole night’s sleep pattern. However, although we conducted experiments on only 10 people, the results confirmed the feasibility of applying our system to night sleep. Second, we examined the stimulation effect on heart signals. Heart rate oscillations interact with other mechanisms, such as the baroreflex or chemoreflex. Grimaldi et al. [29] assessed the effect of acoustic stimulation during sleep on HRV, blood pressure (BP), and cortisol. An enhancement in SWA was associated with a reduction in evening-to-morning variation in cortisol levels and indices of sympathetic activity. However, they did not identify an association between BP changes and SWA enhancement, as observed in HRV and cortisol. Further investigations are required to clarify the physiological effect of stimulation by measuring BP, cortisol, and CO_2_ signals.

Third, we only included healthy people in our study. Our system could be applicable to persons with arrhythmia who have to utilize a pacemaker, a device that generates electrical stimulation and regulates heart rhythm. Our system does not change stimulus BPM by detecting heartbeats in real-time and making contact with the heart directly like a pacemaker, but the methods are similar in that they try to modulate heart rhythm by applying a stimulus. We will evaluate the effectiveness of our system on persons with arrhythmia in a future study.

Fourth, we tested our system for approximately 90 min during napping. Generally, one sleep cycle, i.e., NREM-REM sleep, is completed within 90 min. In night sleep, the sleep cycle is repeated approximately 4–5 times, and we need to evaluate the effect of our system over several sleep cycles. Finally, we could not compare our system performance with those of other stimulation methods. We first developed a closed-loop vibration system and utilized it during naps. Not only are there no studies that apply vibration stimulation during naps, there are only studies that apply other stimulation methods conducted using polysomnography (PSG) during sleep. To solve these issues, we intend to evaluate the proposed system during night sleep by using a PSG test in the future. From the PSG test, we will be able to confirm the changes in sleep stages, which will be scored by sleep technologists, and analyze the changes in brain waves or ANS characteristics in each sleep stage.

## 5. Conclusion

Closed-loop vibration stimulation systems influenced heart rhythm and derived the stabilization of the ANS. A small detuning percent was appropriate for modulating heart rhythm, implicating that an external stimulus closer to the HR has a larger effect on HR modulation. These results suggest that a closed-loop vibration stimulus during sleep could be therapeutic for cardiovascular health, preventing associated diseases. Although various stimulation methods for sleep enhancement have been developed, our system is innovative, as it is unobtrusive and practical for long-term use. We believe that this study can lead to a new strategy for sleep enhancement.

## Figures and Tables

**Table 1:** Results of heart rate variability analysis (Mean ± Standard Deviation).

Variable/Group	Baseline	−3%	−5%	−10%
Mean HR	70.38 ± 6.84	68.44 ± 7.25	67.42 ± 7.67	67.54 ± 3.91
pNN50	7.11 ± 4.20	9.22 ± 7.64	9.52 ± 7.03	11.89 ± 6.25
SDNN	58.41 ± 11.03	56.19 ± 18.36	58.90 ± 18.41	61.01 ± 15.89
RMSSD	38.15 ± 13.97	38.65 ± 17.20	39.95 ± 15.64	46.26 ± 18.12
nLF	0.63 ± 0.10	**0.56 ± 0.10 ^a^**	0.58 ± 0.10	0.55 ± 0.15
nHF	0.37 ± 0.10	**0.44 ± 0.10 ^a^**	0.42 ± 0.10	0.45 ± 0.15
LF/HF	2.30 ± 1.10	**1.84 ± 1.04 ^b^**	1.81 ± 0.83	1.75 ± 1.04

HR—heart rate; pNN50—percentage of successive NN intervals that differ by more than 50 ms; SDNN—standard deviation of the NN intervals; RMSSD—root mean square of the successive NN interval differences; nLF—normalized low-frequency band power; nHF—normalized high-frequency band power; LF/HF—ratio of the low-frequency power to the high-frequency power. Values that were significantly different between the baseline and stimulation conditions are shown in bold. a: *p* < 0.03 between the baseline and −3% stimulation conditions, b: *p* < 0.01 between the baseline and −3% stimulation conditions (Wilcoxon rank-sum test).

**Table 2:** Distribution of heart rate densities (mean ± standard deviation).

Group/Variable		±0.5 BPM	±1 BPM	±2 BPM
**−3%**	Surrogate	0.10 ± 0.02	0.18 ± 0.04	0.35 ± 0.08
	Stim	**0.12 ± 0.04 ^a^**	**0.23 ± 0.07 ^b^**	**0.42 ± 0.12 ^b^**
−5%	Surrogate	0.08 ± 0.01	0.16 ± 0.03	0.31 ± 0.05
	Stim	0.10 ± 0.02	0.17 ± 0.03	0.33 ± 0.05
−10%	Surrogate	0.04 ± 0.01	0.07 ± 0.02	0.14 ± 0.05
	Stim	0.04 ± 0.01	0.07 ± 0.03	0.14 ± 0.04

Values that were significantly different between the baseline and stimulation conditions are shown in bold. a: *p* < 0.01 between the surrogate and −3% stimulation conditions, b: *p* < 0.03 between the surrogate and −3% stimulation conditions (Wilcoxon rank-sum test).

**Table 3:** Summary of the synchronization ratio under each set of stimulation conditions.

	−3%		−5%		−10%	
Subject	Surrogate	Stim	Surrogate	Stim	Surrogate	Stim
1	3.28	5.03	1.37	0.69	1.21	0.74
2	5.15	19.12	3.65	7.76	0.00	1.00
3	0.59	2.97	3.38	9.34	1.55	1.12
4	0.57	10.11	0.00	2.52	0.00	0.00
5	0.65	0.00	0.63	0.00	0.00	0.00
6	1.12	1.26	0.00	0.59	0.00	0.00
7	2.95	3.73	1.21	0.00	0.00	1.05
8	5.23	8.91	1.13	1.17	0.00	0.00
9	3.84	0.56	3.32	1.36	0.00	0.00
10	2.62	0.75	4.63	2.48	0.57	0.67
Average	2.60	5.25	1.93	2.59	0.33	0.46
SD	1.72	5.68	1.58	3.11	0.56	0.48

**Table 4:** Questions about sleep and comfort of the stimulation (mean ± standard deviation).

Question\Group	Baseline	−3%	−5%	−10%
Subjective SOL (min)	15.6 ± 8.3	14.4 ± 10.9	17.2 ± 8.2	12.8 ± 6.3
How was the sleep quality? (0–5, no sleep at all–very good sleep)	3.4 ± 0.8	3.9 ± 1.1	4.1 ± 0.8	4.1 ± 1.2
Felt external stimuli while sleeping (0–5, felt nothing–felt very well)	0.1 ± 0.3	1.0 ± 1.1	0.6 ± 0.8	0.7 ± 0.8
I couldn’t sleep because of the external stimulus (0–5, no–yes)	0.0 ± 0.0	0.4 ± 0.9	0.5 ± 0.7	0.3 ± 0.5

SOL—sleep onset latency, which indicates the time from “lights out” to the first epoch of any sleep stage.
